# The Dynamics of Mastery Motivation and Its Relationship with Self-Concept in Music Education

**DOI:** 10.3390/bs13080667

**Published:** 2023-08-10

**Authors:** Márta Janurik, Tun Zaw Oo, Noémi Kis, Norbert Szabó, Krisztián Józsa

**Affiliations:** 1 Béla Bartók Faculty of Arts, University of Szeged, 6722 Szeged, Hungary; 2 MTA-MATE Early Childhood Research Group, 7400 Kaposvár, Hungary; 3 Institute of Education, Hungarian University of Agriculture and Life Sciences, 7400 Kaposvár, Hungary; 4 Educational Authority, 1055 Budapest, Hungary; 5 Institute of Education, University of Szeged, 6722 Szeged, Hungary

**Keywords:** mastery motivation, self-concept, music education, relationship

## Abstract

Musical education hinges on students’ mastery motivation and self-concept, which are crucial for effective musical learning. Despite the acknowledgement of their individual importance, the relationship between these factors within music education remains unexplored. Hence, this study aimed to investigate the dynamics of mastery motivation (MM) and its relationship with self-concept (SC) in the context of music education. A survey was administered to 139 Hungarian grade 7 students, employing a musical MM questionnaire, a musical SC inquiry, and the collection of demographic information collection. We employed descriptive statistics (IBM SPSS 23), a Rasch analysis (WINSTEPS), and correlational and regression analyses (R programming and SmartPLS4) for data analysis. The findings demonstrated that the utilized instruments were reliable and valid in measuring students’ MM and SC in music education. This study revealed a strong positive correlation (r = 0.778) between students’ MM and SC, with moderate to strong inter-relationships among various subfactors. Furthermore, comparisons unveiled significant disparities in musical MM across school levels, with higher MM and SC observed among female students. Furthermore, gender, a musical family background, and awareness of musical lesson usefulness were predictive factors for both students’ MM and SC within music education. This study provides valuable insights for professionals and policy makers to enhance music education, nurturing students’ musical growth effectively. By understanding the relationship between MM and SC and considering predictive factors, stakeholders can develop strategies that optimize the impact of music education on students’ musical development.

## 1. Introduction

In the modern era, individuals have increasingly recognized the significance of music education, as it possesses the ability to communicate with people through three different voices such as the voice of music itself, a persuasive voice, and an advocacy voice [1]. Consequently, people hold music in high regard and acknowledge the necessity of providing their children with a solid musical education. To provide children with a solid musical foundation, it is crucial to consider the role of children’s mastery motivation (MM) and its related factors.

Within the field of music education, there has been increasing interest in understanding the importance of MM which is crucial in children’s effective musical learning lessons [2]. MM is a form of motivation that refers to the internal drive to attain and enhance one’s abilities without being motivated by external or tangible rewards [3,4]. It is also defined as an individual’s inherent desire that propels and sustains a behavior centered around mastering a difficult task, skill, or competency [5]. Numerous studies have been conducted to investigate MM as a potential factor that explains different aspects of academic accomplishment, student engagement, mental well-being, cognitive abilities, and future career decisions, among others [6,7,8,9]. Consequently, the importance of MM holds true for students across various educational disciplines. Recent research has also shown the importance of motivation for the effective learning of musical students [10,11,12,13]. However, research about MM is very rare in the field of music education.

Another important factor for students’ effective musical learning is their self-concept (SC), which encompasses the amalgamation of how an individual perceives, believes, and constructs self-representations regarding their musical abilities and potential [14,15]. The concept of musical SC revolves around an individual’s realization of their musical abilities, which stems from their interpretation of personal experiences [16]. The musical SC is crucial for students as a distinctive predictor of musical achievement, indicating a link between their abilities and perception of themselves as talented musical students [17]. Several research studies have recognized the significance of musical SC as a crucial socio-cognitive element that governs students’ behaviors, level of effort, and motivation when it comes to practicing and engaging in music learning tasks [14,15,18,19,20,21].

Within the realm of music education, extensive research has emphasized the significance of students’ SC on their musical learning experience, as evident by notable studies conducted by Arens et al. [22], Fiedler and Hasselhorn [2], and Morin et al. [14]. However, despite this wealth of knowledge, exploration into the realm of MM remains scarce. While individual investigations of SC and MM have contributed to our understanding, a comprehensive comprehension of their intricate interaction, the mutual influence they exert upon each other, and the potential variations in these constructs across schools and genders is still lacking within the context of music education. Consequently, there is an inherent need to bridge these gaps and delve deeper into the dynamics between MM and SC, shedding light on their interrelationship and examining their relevance within the unique realm of music education. By undertaking this study, we aim to offer valuable insights into these constructs, uncovering their dynamics and exploring how they interplay with the contextual factors of music education, thus enriching our understanding of these crucial components.

## 2. Literature Review

### 2.1. Self-Determination Theory

Self-determination theory (SDT) is a comprehensive framework that encompasses various aspects of human motivation and the formation of personality [23]. SDT can provide valuable insights into the relationship between MM and SC in music education. According to SDT, individuals have three basic psychological needs such as autonomy, competence, and relatedness [24]. Recent research has explored how these needs interact with musical MM and SC. For instance, one study [25] has shown that when students feel a sense of autonomy in their learning, they tend to develop a stronger SC and self-determined perception. Autonomy, which relates to having a sense of freedom for students in music, is positively related with their musical SC in learning music [26]. Competence, another psychological need, is closely linked to MM in music. Students who perceive themselves as competent and effective are more likely to exhibit strong MM or the desire to improve and develop skills [27]. Higher levels of competence are mostly associated with greater intrinsic motivation and a drive for musical mastery. Therefore, students who feel competent are more likely to strive for musical excellence, which, in turn, can enhance their SC as skilled students. Relatedness, the need for social connections and a sense of belonging, also plays a crucial role in shaping musical SC [23]. Research has shown that students who perceive a supportive and collaborative social environment are more likely to develop a positive SC as talented musical students [17]. 

By examining the relationship between musical MM and musical SC through the lens of SDT, we gain a deeper understanding of the underlying mechanisms that drive students’ MM and shape their SCs in music. While these recent findings provide insights into the connection between SDT and the relationship of MM and SC, further investigation is necessary to unravel the complexities of the relationship and identify potential moderating factors.

### 2.2. Perspectives of Mastery Motivation (MM) and Self-Concept (SC) in Music Education

#### 2.2.1. Mastery Motivation

In 1959, White [28] proposed a nonhomeostatic motive for interacting effectively with the environment and for curious, playful exploration that he dubbed ‘effectance motivation’. This concept of ‘effectance motivation’ inspired Yarrow and colleagues to conduct research that led to our mastery motivation approach [29,30]. Barrett and Morgan [31] then defined MM as a complex psychological drive that motivates individuals to engage with and overcome moderately challenging skills or tasks. What differentiates this approach to motivation from other motivational perspectives is its emphasis on the persistence and determination displayed when facing difficult tasks within specific domains [3,31]. The uniqueness of MM lies not only in its focus on confronting challenging tasks but also in its incorporation of various domains of mastery [32]. Actually, MM encompasses two main indicators or behaviors: (1) instrumental behaviors characterized by persistent efforts towards specific tasks or goals and (2) affective and expressive behaviors such as experiencing pleasure in mastering something and showing anger, frustration, sadness, or shame when faced with challenges [6]. MM drives students to confront and strive to overcome moderate challenges across different domains of development, often by applying diverse strategies to determine which ones are effective [3,6]. MM can vary in different contexts and areas of focus [7,31,32]. Apart from these two broad elements of mastery motivation (instrumental and affective), individuals can exhibit varying levels of mastery motivation across different areas of their development. These areas can be broadly classified as non-social, social, and self-mastery domains [33]. A student’s mastery motivation is multifaceted with domains of self-as-agent, their social context, and their non-social or cognitive aspects [6,34]. Within these domains, more specific areas of mastery can be distinguished, such as mastering social interactions with peers or adults, developing skills in self-initiated motor behavior, achieving mastery over emotions, and overcoming impulsive tendencies [3]. Therefore, we can see that a student’s mastery motivation is classified into two indicators (instrumental and expressive) in three domains (non-social, social, and self-mastery). The current study examines two indicators (instrumental and expressive) of students’ mastery motivation in music education. 

The U.S. National Academy of Science’s report From Neurons to Neighborhoods [9] identified mastery motivation as a key developmental concept, which should be included as part of a child’s assessment. Thus, mastery motivation is an important topic, in part because there is evidence that better mastery motivation leads to better competence and achievement later. That is, children become more competent because of their early persistence at tasks, even if early on they are not highly competent [6]. Jόzsa and Molnár [8] found that the DMQ was more predictive of school grades than IQ and tests of basic skills. More recently, Józsa, Kis, and Barrett [3] found that mastery motivation in preschool children predicted school success in grades 1 and 2.

Within the field of music, Józsa, Kis, and Huang [7] compared MM as the subject-specific mastery motive between Hungarian and Taiwanese students. Their findings indicated that the connection between subject-specific mastery motives becomes progressively weaker as students advance through grades 4 to 10. In this comparative study, Hungarian students demonstrated lower motivation in music than Taiwanese students. Moreover, the MM of Hungarian students decreased significantly between grade 4 and grade 8. From the school subjects under investigation, this decrease was most radical in music among Hungarian students. Taiwanese students, on the other hand, did not demonstrate a significant decrease in their musical MM over the period under investigation. Janurik et al. [35] broke down musical MM into further dimensions. They distinguished between persistence in the acquisition of singing, rhythm reading, music reading, and musical knowledge as well as between musical mastery pleasure and failure in musical activities. These variables are associated with the attitude towards school music lessons. 

The literature lists the studies which examined the relationship between MM and SC. Szenczi, Kis, and Józsa [15] revealed a link between academic SC and MM among grade 6–10 students with learning disabilities. According to their results, cognitive persistence was associated with mathematical SC, general school SC, general self, physical abilities, and the verbal and social facets of SC. Moreover, general SC was found to correlate with cognitive persistence, gross motor persistence, and social persistence with adults. In their large-sample study among Hungarian students, they found moderate to strong correlations between MM and the different facets of SC. 

#### 2.2.2. Self-Concept

According to the most general definition of SC, it is “how one describes oneself” [36], p. 612. Its development is affected by self-perception, self-appraisal, self-representation, self-evaluation, and self-description. The most comprehensive model of SC was developed by Shavelson and colleagues [37]. Their model was the foundation for further research that confirmed the existence of specific facets of SC [38,39,40]. General SC is a hierarchical construct which can be divided into specific layers: learning, social, emotional, and physical SC, which can be further divided into specific areas on a lower level. Layers, as well as their parts, are called domains of SC. Research has confirmed that subject-specific SCs are often not connected to each other or demonstrate only weak connections [41,42,43]. 

Musical SC is a component of general SC, it covers representations with regard to “who I am” in music and “what I can do” in music [44], p. 268. Vispoel’s [45] musical SC model builds on previous research on SC and covers both general and specific areas. His research confirmed that musical SC is part of a SC hierarchy involving a hierarchically superordinate artistic domain and hierarchically subordinate components (music composition, instrument playing, music reading, singing, listening, and dancing). Results suggest that facets of musical SC are connected to each other; however, singing and dancing SCs seem to be more distinct from the other facets of musical SC [14]. A positive musical SC was found to be linked to positive expectations with regard to musical achievement and predicted more effort and higher achievement in musical activities [46]. Vispoel [47] suggests that overall perceptions of one’s musical abilities are dominated by perceptions of skills in instrumental performance and auditory cognition. Music reading, singing, and instrumental play were identified as key components in adolescents. Instrumental play was found to be the most significant factor in musical SC, and perceived low skills in instrumental performance were combined with a lower self-esteem. Vispoel’s [45] results support previous findings with regard to the role of domain importance effects in musical SC [39,47,48]. This implies that global self-esteem has a weaker relationship with musical SC if an individual considers being educated in music less important. However, when one is keen on being musically educated, the relationship between global self-esteem and musical SC is stronger [49]. Research has also confirmed that musical SC is significantly correlated with intrinsic motivation and internal attributions as well [46,50]. Musical SC was found to be relatively stable among elementary school students; however, competition, which is considered an external motivator, and evaluation may have a positive impact on SC [51]. Based on Schmidt’s [20] research, musical SC is moderately correlated with mastery orientation, intrinsic orientation, and individual orientation as well. Within the three factors identified by Schmidt [20], musical SC demonstrated similar factor loadings for individual orientation and learning/task orientation. The research of Schmidt [20] as well as that of Marsh et al. [52] confirms that musical SC is linked to intrinsic motivation.

Based on the research of Shavelson et al. [37], Spychiger, Gruber, and Olbertz [21] proposed a multi-dimensional model of SC which assumes that not only those musically active but all of us have a musical SC. Within this musical SC, they distinguish between non-academic components—emotional, social, physical, cognitive, and spiritual representations related to music—and ideal musical self, as well as an academic component which concerns musical ability. In their view, this model provides a better understanding of one’s musical SC than just looking at perceptions about one’s musical ability. According to their results, the cognitive component of musical SC is strongest in professional musicians and music workers, while the spiritual component was found to be strongest in “leisure” musicians. The cognitive, social, and spiritual components all demonstrated the lowest mean values in music listeners. MUSCI_youth was used by Fiedler and Spychiger [53] among German high school students.

### 2.3. Brief Oveview of Hungarian Music Education

The aim of music education in Hungarian elementary schools is to cultivate students’ personality in a diverse way through music. In line with the Kodaly Method, the curriculum establishes activity-centered education which relies on musical experiences as the primary aim [54]. Among musical activities, singing is given priority in school music lessons. Singing is used as a method for developing musical skills as well as inner hearing, learning about the history of music, enhancing creativity, and strengthening the community. Rhythmic activities (clapping, tapping, simple rhythmic instruments, or “body instruments”) provide diverse opportunities for the acquisition and practice of rhythmic skills and rhythmic knowledge [55]. Students start to learn musical notation at the age of 6, in first grade. Musical cognitive processes and forming of mental representations, which are needed for music reading, are facilitated through enhancing singing skills and musical perception, the foundation of which are singing and rhythmic activities [56,57].

### 2.4. The Context of Current Study

The existing body of literature demonstrates a considerable number of studies focused on students’ self-perception in the realm of musical education, for instance in these studies [36,37,38,39,40,41,42,43]. However, when it comes to MM in music education, only a limited number of studies have been conducted, for instance, those by Janurik et al. [35] and Józsa et al. [7]. According to SDT, a valuable perspective is suggested by examining the relationship between students’ MM and SC in the context of music education. By gaining an understanding of Hungarian Music Education, it becomes evident that there is an opportunity to carry out a survey study to explore the connection between students’ MM and SC in the field of music education. Therefore, this research aimed to investigate the relationship between MM in school music lessons and musical SCs, including its components. 

Based on the studies by Józsa et al. [7] and Calchei et al. [5], we learned that age and gender differences can have impacts students’ musical MM. Regarding the musical SC, Shavelson et al. [37] and Spychiger et al. [21] mentioned that non-academic components such as students’ emotional, social, physical, cognitive, and spiritual representations can have relationships with their musical SCs and abilities. Therefore, this study additionally explored the potential influence of background variables such as elective instrumental training, duration, musical family background, music grade, gender, and students’ perspectives on the usefulness of school music lessons on musical MM and SC. This study focused on addressing the following research questions in accordance with its research aims.

(1) What relationship can be established between musical MM and musical SC?
(2) How do students’ musical MM and SC differ in different school levels and genders?
(3) To what extent do the background variables predict the musical MM and SC?

## 3. Materials and Methods

### 3.1. Participants

Participants included 139 grade 7 (age M = 13.51, SD = 0.68) Hungarian students (68 boys and 71 girls) from eight classes of four elementary schools in a city in the southern part of Hungary. Data collection took place at the end of the school year. Parents were informed about the objectives and details of the research, and they provided their written consent.

### 3.2. Instruments

#### 3.2.1. Musical MM Questionnaire

Musical MM was examined with the method of Janurik et al. [35]. Its five-point Likert-type items rely on the main musical activities of Hungarian school music lessons. The consolidated indicators of MM were created based on the scales of musical MM: (1) instrumental component (music learning persistence): rhythm acquisition, singing acquisition, acquisition of music reading, and musical knowledge (18 items) and (2) expressive component: musical mastery pleasure and negative reaction to musical failure (6 items). The consolidated indicator of musical MM was created based on the instrumental and expressive components (24 items). It is as below with related examples.

Instrumental Component:

(a) Rhythm acquisition, e.g., “I practice rhythm clapping in order to be good at it.” (6 items);
(b) Singing acquisition, e.g., “I do my best to sing beautifully.” (6 items);
(c) Acquisition of music reading and musical knowledge, e.g., “I engage in a music reading or a musical knowledge-related task until I can master it.” (6 items).

Expressive Component:

(d) Musical mastery pleasure, e.g., “I’m happy if I manage to read the sheet music of a song, or if I manage to understand something related to music.” (3 items);
(e) Negative reaction to musical failure, e.g., “If I cannot sing a song beautifully, I don’t even start singing it, or I stop singing it.” (3 items).

#### 3.2.2. Musical SC Inquiry

Fiedler and Spychiger’s [53] Adjusted Musical SC Inquiry (MUSCI, youth) was used to examine musical SC. The method examines musical SC as a multi-dimensional construct which, on the one hand, consists of non-academic components, entailing emotional, social, physical, cognitive, and spiritual representations, and on the other hand, has an academic component which concerns musical ability. Participants indicated their level of agreement with the items on a 4-point Likert-scale (28 items). Subscales and samples of MUSCI are the following:

(a) Mood Management, e.g., “Music relieves me from daily routine.” (6 items);
(b) Community, e.g., “I easily socialize by the means of music.” (4 items);
(c) Musical Ability, e.g., “My musical ability is above average.” (5 items);
(d) Movement and Dance, e.g., “I easily move to the rhythm of music.” (4 items);
(e) Ideal Musical Self, e.g., “I would like to have higher musicianship.” (5 items);
(f) Adaptive Musical Self, e.g., “My physical reaction to music is different from what it was formerly.” (4 items).

### 3.3. Background Variables

The following areas were investigated: elective instrumental training (1 item); duration of elective instrumental training (1 item); views on the usefulness of school music lessons (2 items, Cronbach’s ᾳ = 0.76); musical family background: (1) Do you have any musical instrument at home? (2) Do you have any family member who is a musician? (3) Does your mother sing at home? (4) Does your father sing at home? (5) Can your mother play any musical instrument? (6) Can your father play any musical instrument? Cronbach’s ᾳ = 0.60); mother’s level of education (1 item); and music grade (1 item). Students provided the grades they were awarded in music. School grades in Hungary range from 1 to 5, with 1 marking the poorest academic progress and 5 marking excellent academic progress.

### 3.4. Procedure

Students filled out the musical MM questionnaire during a regular music lesson. The musical SC questionnaire and the background questionnaire were filled out during another lesson. The musical SC and the musical MM questionnaires took 20 min each to fill out, while the background questionnaire took 10 min. The information concerning mothers’ level of education was provided by the teachers.

### 3.5. Data Analysis

We examined the descriptive statistics using IBM SPSS version 22.0. To assess the reliability of the instruments, a composite reliability (CR) value above 0.70 and an average of variance extracted (AVE) value above 0.50 were recommended [58]. The adequacy of the participants for data analysis was also assessed by the Kaiser–Meyer–Olkin (KMO) value which was greater than 0.70 [59]. In order to confirm the constructs of both the musical MM and SC instruments, we conducted a confirmatory factor analysis (CFA) using the criteria suggested by Kline [59]. These criteria include the comparative fit index, CFI ≥ 0.90; root mean square error of approximation, RMSEA ≤ 0.07; and standardized root mean square residual, SRMR ≤ 0.08. The characteristics of the measuring instruments (test characteristics) were analyzed using WINSTEPS Rasch Analysis [60]. To investigate the relationship between students’ musical MM and SC, SmartPLS4 software with covariance-based structural equation modeling (CB-SEM) and R Studio were utilized. The recommended correlation values, as defined by Gliner et al. [61], were as follows: 0–0.3 (low), 0.3–0.5 (moderate), and above 0.5 (strong). To compare musical MM and SC based on school levels and gender, ANOVA and *t*-tests were employed, and the results were visualized using R programming with ggplot2, ggpairs, and dplyr packages [62]. To predict the impact of background variables on students’ musical MM and SC, Partial Least Square Structural Equation Modeling (PLS-SEM) was conducted using SmartPLS4 [63]. Path analysis based on partial regressions was also performed. The model fit was assessed using the following fit indices with SmartPLS4 software: Chi-square test, normed fit index (NFI), and Standard Root Mean Square Residual (SRMR). An NFI value ≥ 0.90 indicated a good fit, while an SRMR value ≤ 0.08 suggested an appropriate fit. The d-value in Unweighted Least Square (d-ULS) and the d-value in Geodesic Distance (d-G) were used to evaluate model fit, with smaller d-values closer to zero indicating a better fit [64]. 

## 4. Results

### 4.1. Descriptive Statistics

As for the background variables, 28 students (20.1% of the sample) used to learn or still learned to play a musical instrument at the time of data collection. Out of them, nine students learned it for 1 year (6.5%), three students for 2 years (10.3%), five students for 3 years (2.2%), two students for 4 years (1.4%), seven students for 5 years (5.0%), two students for 6 years (1.4%), and one student learned to play a musical instrument for 7 years (0.4%). The average value for the usefulness of school music lessons was M = 2.55 (Minimum = 1, Maximum = 4, SD = 0.83). The average music grade was M = 4.24 (SD = 0.89). According to musical family background variables, 25% of students had a distant family member who was a musician, 51% of the students had a musical instrument in their homes, 37% of mothers and 22% of fathers regularly sang at home, and 9% of mothers and 8% of fathers could play a musical instrument. 

### 4.2. Reliability and Validity of Instruments

In order to assess the construct validity of both instruments, we examined convergent and discriminant validities. Convergent validity was evaluated through measures such as internal consistency reliability (Cronbach’s alpha), composite reliability (CR), and average variance extracted (AVE), and their respective recommended values were confirmed as indicated by (*) [61]. To begin, we assessed the adequacy of the sample by calculating the Kaiser–Meyer–Olkin (KMO) value. The musical MM questionnaire yielded a KMO value of 0.902, while the musical SC inquiry obtained a KMO value of 0.859. These values surpassed the recommended threshold of 0.70 established by Kline [59], indicating satisfactory sampling adequacy. Convergent validity, as demonstrated by internal consistency values (Cronbach’s alpha, CR, and AVE), was found to be good for both instruments, as depicted in Table 1 and Table 2. 

**Figure 1:**
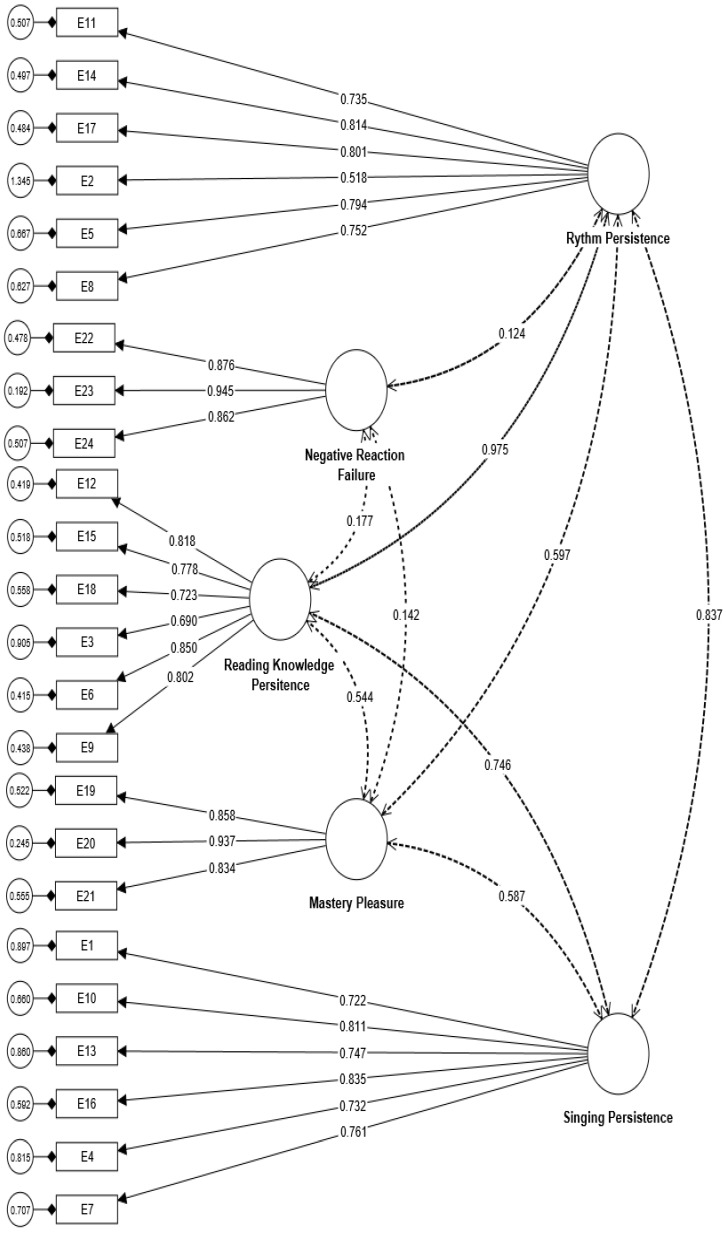
CFA model for musical mastery motivation.

**Figure 2:**
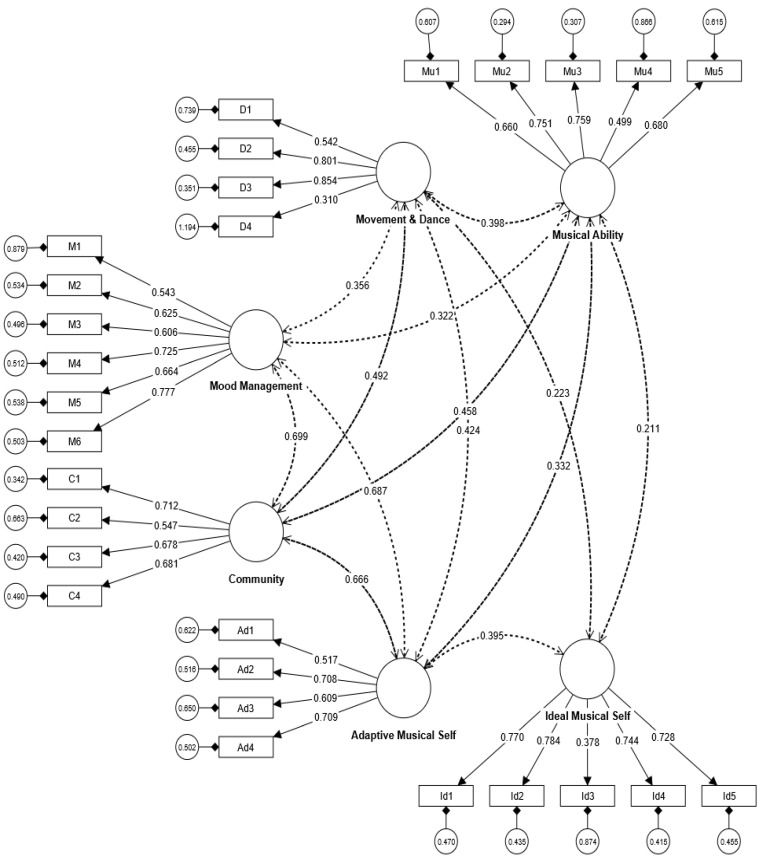
CFA model for musical self-concept.

Consequently, the study suggests that the variance in the total amount of true scores is relatively equivalent to the variance in the total scale score of both instruments. Furthermore, the measured values of all factors for both instruments (five factors for the musical MM questionnaire and six factors for the musical SC inquiry) aligned with the recommended values indicated by asterisks (*) in Table 1 and Table 2. The factor loadings for all factors are also above 0.40. In order to confirm the underlying dimensions that contribute to the measured variables of musical MM and SC assessments, we conducted separate CFA models with the help of CB-SEM in SmartPLS4 software. The CFA model for musical MM demonstrated favorable fit indices (Chi-square/df = 2.69, CFI = 0.90, SRMR = 0.04, RMSEA = 0.06). Similarly, the CFA model for musical SC assessment also exhibited good fit indices, including Chi-square/df = 1.96, CFI = 0.92, SRMR = 0.05, and RMSEA = 0.06 [64]. The CFA models for both musical MM and SC assessments are visually presented in Figure 1 and Figure 2, respectively. There are significant positive correlations among factors of both instruments of musical MM and SC. Thus, the study concludes that all items and factors in both instruments are closely related. Based on the findings of convergent and discriminant validities, the study also interprets that both instruments exhibit satisfactory construct validity.

To evaluate discriminant validity, we analyzed the square root of AVE and component correlations of both versions of the instruments, as presented in Table 3. The square roots of AVE values (*) from the instruments were found to be higher compared to other component correlations. This outcome clearly demonstrates the effectiveness of the instruments in accurately measuring the intended concept, which in this case is the assessment of students’ musical MM and SC. Accordingly, the study suggests that both instruments possess good discriminant validity when assessing students’ musical MM and SC. 

**Figure 3:**
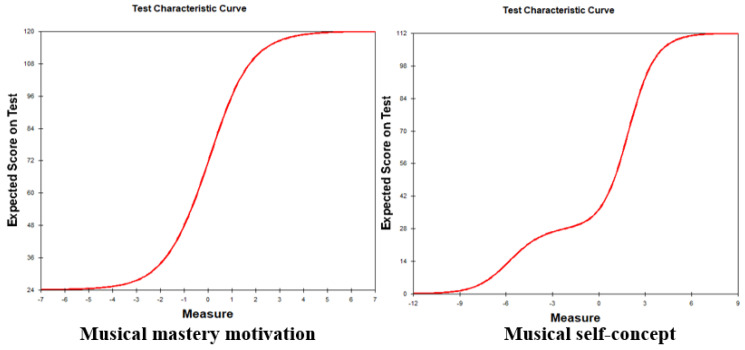
Distribution of the assessments of musical MM and SC.

In order to examine the distribution of student responses in the musical MM and SC questionnaires, we assessed their normality measures. The mean values and standard deviations of the instruments were 2.63 and 0.913 for MM, and 2.28 and 0.562 for SC, respectively. Normality measures were employed to evaluate the test normality for both instruments. To determine severe non-normality, we referred to the guidelines proposed by Kline [59]. According to these guidelines, a severe violation of the normality assumption is indicated by Skewness (Sk) values greater than 3 and Kurtosis (K) values exceeding 10. The results indicated that both the musical MM instrument (Sk = 0.41, K = −0.78) and the musical SC instrument (Sk = 0.06, K = −0.92) displayed a normal distribution when measuring students’ musical MM and SC [64]. Figure 3 illustrates the test characteristic curves of both instruments. These curves describe the relationship between the expected scores and ability measures, indicating for both instruments that students’ abilities increased with higher test scores in musical MM and SC. The finding suggests that the distribution of student responses in both instruments is approximately normal, and the test characteristic curves demonstrate a positive correlation between expected scores and the corresponding ability measures.

### 4.3. Addressing RQ1

The first research question aimed to investigate the relationship between students’ musical MM and musical SC.

**Figure 4:**
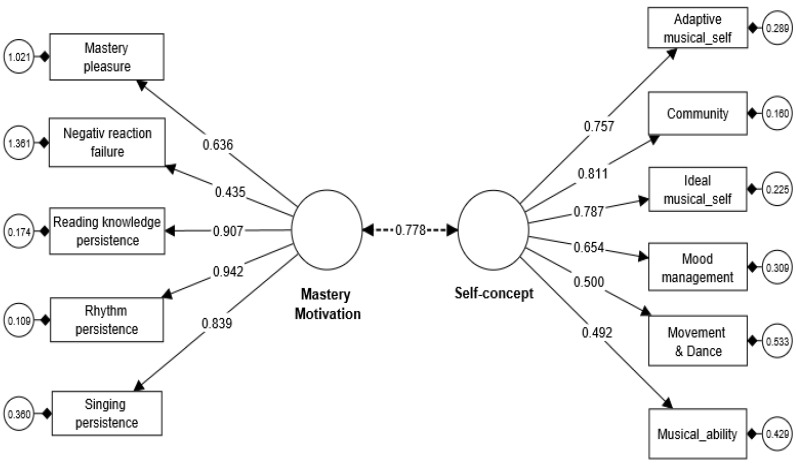
Correlation between musical MM and SC.

To analyze this relationship, we first employed the SmartPLS4 (CB-SEM) procedure and conducted a bootstrapping procedure. The results of the analysis revealed a strong positive relationship between students’ musical MM and their musical SC, as indicated by a correlation coefficient of 0.778 with good fit indices (Chi-square/df = 2.48, CFI = 0.92, SRMR = 0.06, RMSEA = 0.04). This correlation coefficient of 0.778 signifies the strength and direction of the relationship between the two sets of variables, suggesting a substantial positive correlation between musical MM and musical SC (see Figure 4). This finding suggests that as students’ MM towards musical skills increases, their perception and belief in their own musical abilities (musical SC) also tend to improve. It is important to note that a bootstrapping procedure, with 500 subsamples and a significance level of 0.05, was utilized to assess the significance of the correlation coefficient and establish its robustness. The percentile bootstrap method was used for the confidence interval estimation. Based on the results obtained from the canonical correlation analysis, it can be concluded that there is a strong positive relationship between students’ musical MM and their musical SC. 

**Figure 5:**
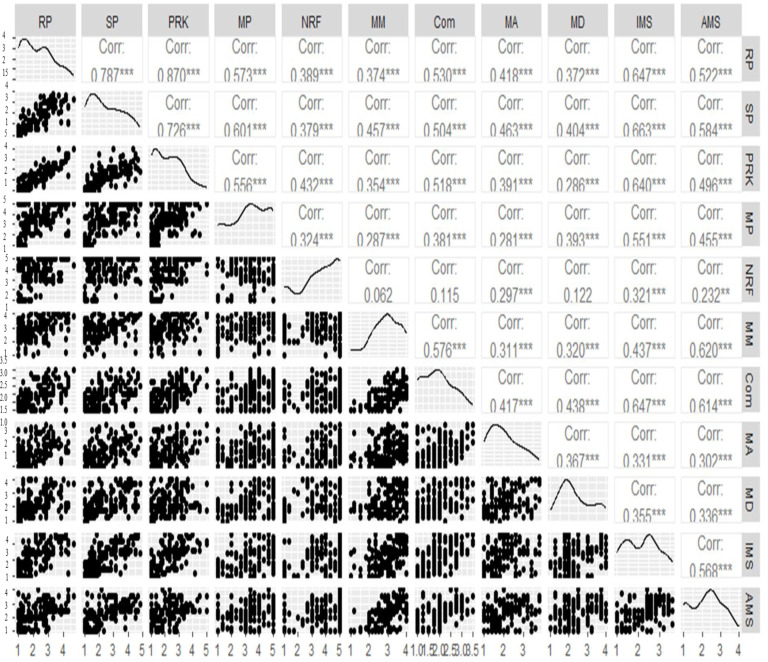
Correlations among different factors of instruments. Note: ** *p* < 0.01, *** *p* < 0.001, MM (mood management), Com (community), MA (musical ability), MD (movement and dance), IMS (ideal musical self), AMS (adaptive musical self) for the musical SC inquiry; RP (rhythmic persistence), SP (singing persistence), PRK (musical reading and knowledge acquisition), MP (musical pleasure), NRF (negative reaction to failure) for the musical MM inquiry.

In the second step of our analysis, we employed the R programming language, specifically using the ‘correlogram with ggpairs’ function, to explore the intercorrelations among different factors present in the two instruments. Figure 5 depicts the complete correlations between the various constructs of the two instruments through scatter plots and Pearson correlations. The strengths of the correlations were interpreted based on the following thresholds: correlations below 0.3 were considered low, correlations between 0.3 and 0.5 were deemed moderate, and correlations above 0.5 were considered strong. These threshold values were suggested by Gliner et al. [61]. The analysis revealed five significant factors in the musical MM questionnaire: rhythmic persistence, singing persistence, musical reading and knowledge acquisition, musical pleasure, and negative reaction to failures for the musical MM inquiry. Additionally, six factors were found in the musical SC inquiry: mood management, community, musical ability, movement and dance, ideal musical self, and adaptive musical self. With the exception of the factor “negative reaction to failure” in the musical MM questionnaire, all other factors in both instruments exhibited moderate to strong relationships with each other. This implies that students’ musical MM is strongly associated with each factor of both instruments. Interestingly, the factor “negative reaction to failure” in the musical SC inquiry displayed low correlations with the other factors (r = 0.06, 0.11, 0.29, 0.12, 0.32, and 0.23, * *p* < 0.05), suggesting that if students have a lower negative reaction to failures in terms of musical MM, they are more likely to have a higher musical SC. For the clarity of the relationship between the musical MM and SC, we also described the correlation coefficients in Table 4.

Furthermore, we examined how students’ background variables (such as gender, music grade, elective instrumental training, duration of instrumental training, awareness of the usefulness of music lessons, and music family background) were related to their musical MM and SC. The correlation coefficients varied from 0.112 to 0.609, indicating weak to moderate correlations (Table 5). The results indicate that these variables do not provide multicollinearity problems, suggesting further analyses of predicting MM and SC in music education. By examining the correlations between the background variables and the musical MM and SC, we gain valuable insights into the potential factors that may influence students’ musical development. For instance, the moderate correlation observed between the usefulness of music lessons and musical MM implies that students’ awareness of the usefulness of music lessons may positively impact students’ MM on learning musical subjects. Furthermore, the weak correlations identified in some cases, such as between music grade and musical MM and SC, suggest that these particular background variables may have limited influence on students’ musical MM or SC. This knowledge helps us focus our attention on other potential factors that might play a more significant role in shaping musical MM and SC.

The research questions examined the variations in students’ musical MM and SC across different school levels and genders. The investigation involved comparing schools and genders in four main aspects: (1) comparing students’ musical MM among different schools, (2) comparing students’ musical SC among different schools, (3) comparing students’ musical MM based on gender, and (4) comparing students’ musical SC based on gender. To analyze the data, we utilized R software along with the ggplot2 and dplyr packages, ANOVA for comparing different schools, and t-tests for comparing different genders.

**Figure 6:**
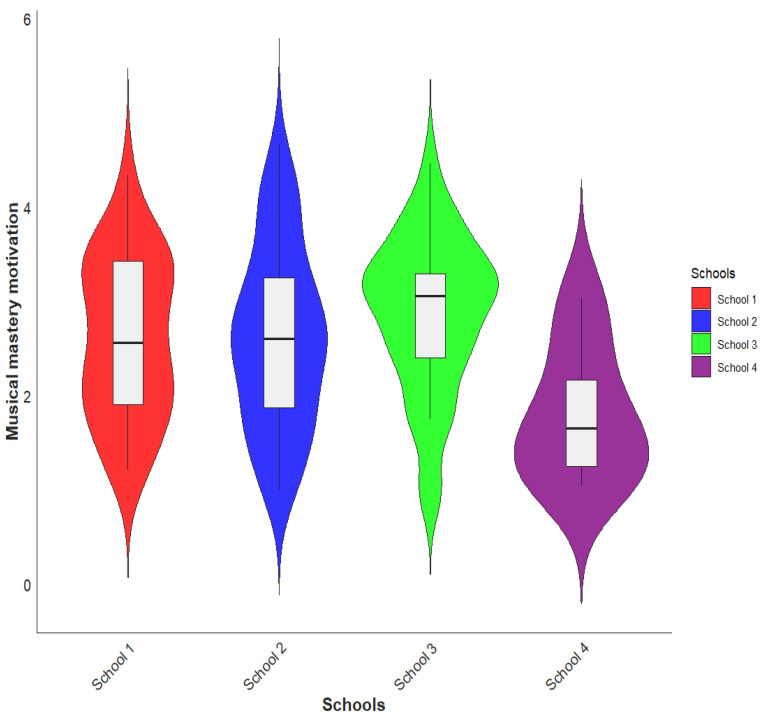
The comparison of students’ musical MM by the schools.

Initially, we conducted a comparison of musical MM among different school levels. We analyzed the data using an ANOVA. The findings revealed distinct variations in motivation levels across the four schools (* *p* < 0.05, F = 2.82, df = 3, between groups). Notably, students from School 3 displayed the highest level of musical MM (M = 3.12, SD = 0.82) among all schools. Following closely behind was School 2 (M = 2.61, SD = 0.85), then School 1 (M = 2.60, SD = 0.95), and lastly, School 4 (M = 1.80, SD = 0.73). Figure 6 illustrates the comparison of musical MM by school level in a graphical representation. Based on the findings, it can be interpreted that students from School 3 exhibited the highest level of musical MM compared to the other schools included in the study. This suggests that the learning environment, teaching methods, and availability of music classes or extracurricular activities at School 3 may be particularly effective in fostering students’ enthusiasm and dedication towards MM in music education. On the other hand, students from School 4 demonstrated the lowest level of musical MMs among the four schools. This could indicate a potential need for support or interventions in promoting students’ MM and engagement in music-related activities at School 4. Furthermore, the differences in MM across schools imply that various factors, such as gender, parent’s education levels, music grades, resources, or overall musical culture, may contribute to variations in students’ MM for music education [35].

**Figure 7:**
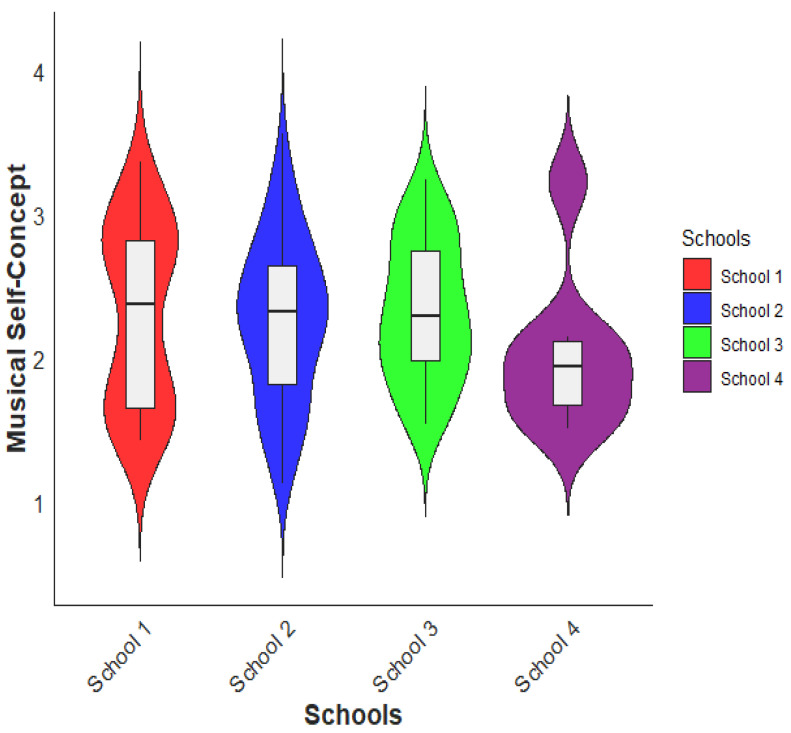
Comparison of students’ musical SC by schools.

Next, we compared musical SC among different school levels by an ANOVA. No significant differences were found between the four schools (* *p* > 0.05, F = 5.27, df = 3, between groups). Notably, students from School 1 displayed the highest level of musical SC (M = 2.32, SD = 0.487) among the four schools in this study. They were closely followed by students from School 2 (M = 2.30, SD = 0.62), then students from School 3 (M= 2.26, SD = 0.56), and finally students from School 4 (M = 1.84, SD = 0.57). The comparison of musical SC by school level is visually depicted in Figure 5, providing a clear illustration of the differences. The findings suggest that, although there were no significant differences between the schools as a whole, there were variations in the levels of musical SC among individual schools depicted in Figure 7. Students attending School 1 possess a stronger sense of self-perception and confidence in their musical abilities compared to students from other schools. Similarly, students at Schools 2 and 3 also exhibit relatively high levels of musical SC. Conversely, students from School 4 demonstrated a comparatively lower level of musical SC. 

**Figure 8:**
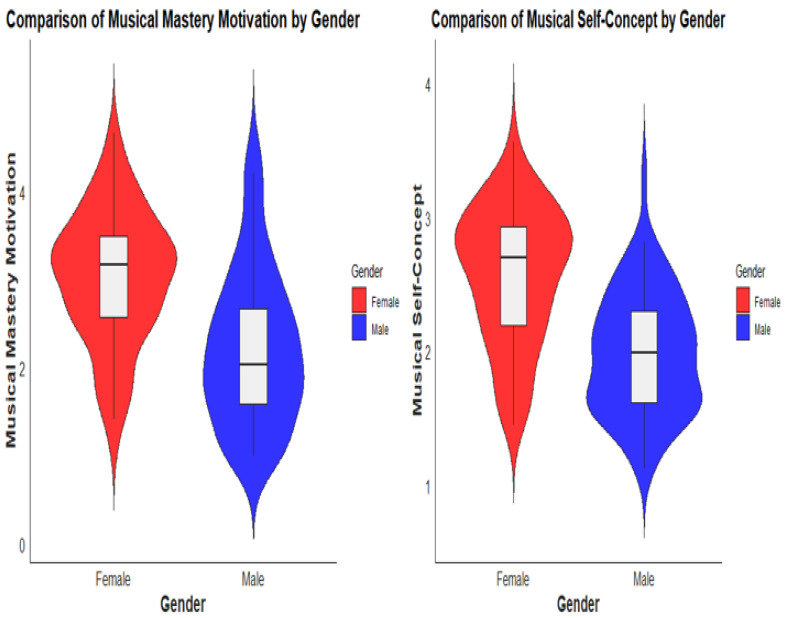
Comparison of musical MM and SC by gender.

Then, in the comparison of students’ MM by gender, we used an independent sample t-test to compare the differences between male and female students. The results indicate that there was a significant difference in MM between male and female students (*** *p* < 0.001). This means that, on average, female students from the sample had higher levels of MM compared to male students. The mean MM score for female students 3.21, with a standard deviation of 0.91. This suggests that, on average, female students in the study exhibited a relatively higher potential for MM. In contrast, the mean MM for male students was 2.22, with a standard deviation of 0.88. This indicates that male students generally had a lower level of MM compared to their female counterparts (see Figure 8). However, it is important to note that this does not imply that all male students had lower MM, as there is variation within each gender group. The significant difference in MM between male and female students suggests that there may be gender-related factors influencing students’ motivation levels. These factors could include societal expectations, cultural norms, or individual differences. 

Similarly, when examining the musical SC of students based on gender, we found a significant difference. In our sample, female students had notably higher levels of musical SC compared to male students (M = 1.98, SD = 0.45 for males; M = 2.56, SD = 0.50 for females, *p* < 0.001) (see Figure 8). The findings suggest a gender disparity in musical SC within the sample. Female students demonstrated a stronger sense of confidence and positive perception regarding their musical abilities and identity compared to male students. This highlights the importance of considering gender differences in musical SC and may have implications for fostering music education and engagement strategies that address these disciplines. 

### 4.4. Addressing RQ3

**Figure 9:**
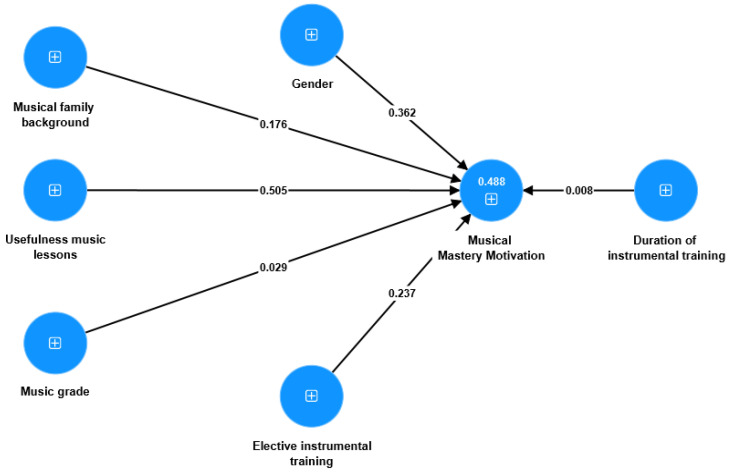
Explanatory power of background variables in students’ musical MM.

This research question aimed to explore how background variables predict musical MM and SC. To analyze the relationships, we utilized the Partial Least Square Structural Equation Modeling (PLS-SEM) approach in SmartPLS4 software. We explored various models that considered both direct and indirect effects among six predictors (gender, musical family background, usefulness of music lessons, music grade, elective instrumental training, and duration of instrumental training) and their impact on students’ musical MM. However, none of the investigated models showed a satisfactory fit. Finally, after thorough investigations, we derived the final model with only direct effects on musical MM by these six predictors (Figure 9). Additionally, the model demonstrated significant relationships between variables and musical MM, and proved to be the most optimal. The results indicated that the model provided a relatively good fit, with the following estimations: SRMR = 0.06, d-ULS (d-value in Unweighted Least Square) = 0.117, d-G (d-value in Geodesic) = 0.111, χ^2^ = 119.30, and NFI (Normed Fit Index) = 0.91. The model revealed that among the six predictors, only three variables (gender, musical family background, and knowledge of the usefulness of music lessons) exhibited significant direct effects. These predictors accounted for approximately 48.8% of students’ musical MM. Specifically, gender played a significant role (β = 0.362, * *p* < 0.05), musical family background had a significant impact (β = 0.176, * *p* < 0.05), and awareness of the usefulness of music lessons showed a substantial effect (β = 0.505, *** *p* < 0.001) on students’ musical MM.

**Figure 10:**
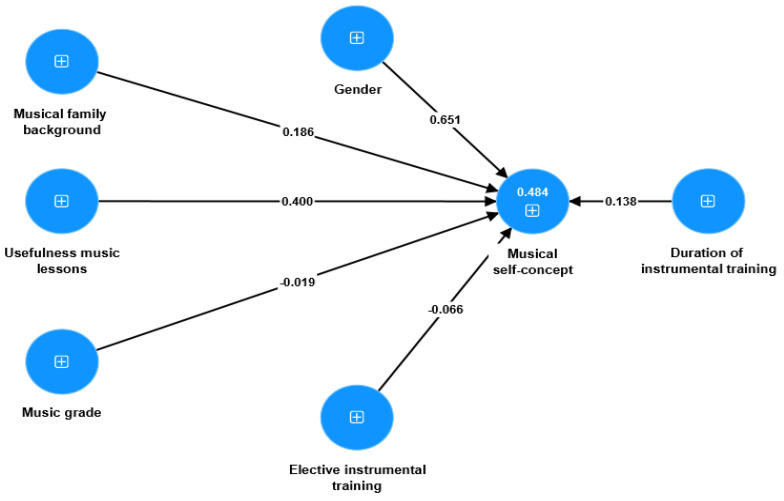
Explanatory power of background variables in students’ musical SC.

Additionally, we investigated the influence of various background factors on students’ musical SC by employing different models (with both direct and indirect effects on musical SC). However, neither of these models had a satisfactory fit. Ultimately, the final model demonstrated a satisfactory fit with acceptable values of fit indexes: SRMR = 0.06, d-ULS = 0.12, d-G = 0.33, χ^2^ = 409.93, and NFI = 0.95 [60]. The findings from the regression analyses showed that only three background variables, namely gender (β = 0.65, *** *p* < 0.001), musical family background (β = 0.18, ** *p* < 0.01), and awareness of the usefulness of music lessons (β = 0.40, * *p* < 0.05), exhibited significant predictive effects on students’ SC in music education (see Figure 10). Other variables such as music grade, elective instrumental training, and duration of instrumental training were found to have no significant effects on students’ musical SC. This suggests that these specific factors may not be strong determinants of how students perceive themselves in the context of music education. 

## 5. Discussion

This study aimed to examine the dynamics between MM and SC within the context of music education. Therefore, we mainly investigated the relationship between musical MM and musical SC in the context of school music lessons. Additionally, we investigated potential differences in musical MM and SC among students based on their school levels and genders. Furthermore, by considering background variables such as views on the usefulness of school music lessons, elective music learning, the duration of elective music learning, musical family background, gender, and music grade, we are able to assess the extent to which these variables predict students’ musical MM and musical SC. This study first confirmed the reliability and validity of the instruments for data analysis for addressing the research questions.

In the investigation of the relationship between musical MM and musical SC, the findings of our analysis revealed a strong positive relationship between these two constructs. A correlation coefficient of 0.778 (* *p* < 0.05) indicated a substantial positive correlation between these two variables. This result suggests that as students’ MM towards musical skills increases, their perception and belief in their own musical abilities (musical SC) also tend to improve. These findings align with previous research emphasizing the positive relationship between MM and SC in school achievement among school-aged children with learning disabilities [15,37]. To assess the robustness of the correlation coefficient, a bootstrapping procedure with 500 subsamples was conducted, and a significance level of 0.05 was employed. The use of bootstrapping provided confidence in the reliability and significance of the correlation coefficient [64]. This approach strengthens the validity of our findings, indicating that the observed relationship between musical MM and musical SC is not a chance occurrence. 

In the second step of our analysis, the intercorrelations among different factors within the two instruments were explored using R programming language. The results revealed significant factors in the musical MM questionnaire, including rhythmic persistence, signing persistence, musical reading and knowledge acquisition, musical pleasure, and negative reaction to failures. Likewise, significant factors emerged within the musical SC questionnaire, involving mood management, community, musical ability, movement and dance, ideal musical self, and adaptive musical self. These factors displayed moderate to strong relationships with each other, indicating a cohesive structure within the constructs of musical MM and musical SC. These findings are consistent with previous studies highlighting the multidimensional nature of both constructs and the interrelatedness of their subcomponents [21,46,50,65]. Notably, the factor “negative reaction to failure” in this musical SC inquiry exhibited low correlations with failure in terms of musical MM and is more likely to possess a higher musical SC. This finding emphasizes the importance of cultivating a positive attitude towards failure as it can contribute to the development of a stronger musical SC or school success [66]. In this study, we also found that the intercorrelations among subfactors have moderate to strong relationships with each other in both instruments. This finding is opposed to previous findings that demonstrated only weak connections among subject-specific SCs [41,42,43]. The reason for strong interrelationships among the subfactors within our musical SC questionnaire may be attributed to the confirmation of instrument reliability and validity specifically in the context of musical education, rather than encompassing all subject-specific fields. Moreover, in the analysis of correlation of students’ background variables with their musical MM and SC, the results showed both weak and moderate correlations. However, an examination of these correlations sheds light on the complex interplay between various factors affecting students’ musical development. These findings contribute to the understanding of how different aspects, such as gender, music grade, elective instrumental training, duration of instrumental training, awareness of the usefulness of music lessons, and music family background, may impact students’ musical MM and SC in the context of music education. This finding aligns with previous research [16,22] that also found a positive influence of gender on students’ musical self-concept. However, the current study introduces novel findings regarding the association between students’ background variables, such as elective instrumental training, duration of instrumental training, musical family background, and music grades, with their musical mastery motivation in the context of music education. However, it is important to note that this study, in contrast to previously cited papers, focused on mandatory music education which is part of the compulsory school curriculum. In addition, we also emphasize that the Hungarian music education in schools is primarily based on singing activities; instrument learning is not included in the curriculum Nevertheless, these new insights suggest the need for further investigation into their relationship in further studies. 

Regarding the comparison of musical MM among different school levels, the findings indicated distinct variations in motivation levels across the four schools. Students from School 3 exhibited the highest level of musical MM, followed by Schools 2 and 1. In contrast, students from School 4 displayed the lowest level of musical MM. When examining the comparison of musical SC among different school levels, no significant difference was found between the four schools on the whole. However, variations were observed in the levels of musical SC among individual schools. In terms of gender differences, significant variations were observed in both musical MM and musical SC. Female students exhibited higher levels of MM and musical SC compared to male students. These findings suggest that gender-related factors may influence students’ motivation levels and their perception of musical abilities. Societal expectations, cultural norms, and individual differences could all contribute to these disparities [67,68]. The results highlight the significance of considering gender differences when designing music education programs and strategies to foster motivation and SC in students [15,35]. Moreover, it is important to consider that Hungarian music education focuses primarily on singing musical activities; instrumental learning is not part of the curriculum. Sanders and Browne [69] suggest that enjoyable music activities may have a positive impact on musical SC. We propose that it works in both ways, however; that is, high musical SC encourages students to seek out enjoyable, challenging music-making experiences, which is the basis of MM.

To explore the predictive role of background variables on students’ musical MM and musical SC, we employed the PLS-SEM approach and analyzed the relationships between six predictors and their impact on students’ MM and SC. Our findings revealed that gender, musical family background, and awareness of the usefulness of music lessons significantly influenced students’ musical MM. These predictors collectively accounted for nearly half of the variation in students’ motivation. Notably, gender differences played a significant role, indicating the influence of gender on students’ motivation in music education. This finding is in line with Somers et al.’s [67] study focusing the importance of gender difference in affecting students’ MM. In terms of SC, gender, musical family background, and awareness of the usefulness of music lessons were found to be significant predictors. This result also aligns with a previous study [17] focusing the importance of musical family background in encouraging students’ SC of taking musical learning courses. Other factors, such as music grade, elective instrumental training, and duration of instrumental training, did not show significant effects on students’ SC. This discovery contradicts earlier findings concerning after-school and elective music education within schools, which had previously affirmed the significance of musical self-perception in both music learning and the ability to persevere in music education [14,44,48,70,71]. The possible reason may be that SC is influenced by individual differences, and the impact of these factors on SC may vary greatly among individuals, making it challenging to detect significant effects at a group level. Moreover, other variables or mediating factors not included in this study may have played a role in shaping SC, such as social support, teacher–student relationships, or extracurricular musical experiences. However, our findings are consistent with previous research [17,66,67,68,69,72], which also identified the significance of gender and family background in shaping students’ musical MM and musical SC.

It is essential to acknowledge some limitations of the study. First, the research was conducted within a specific educational context, which may limit the generalizability of the findings to other settings. Despite the relatively small sample size in this study, the assessed adequacy of participants through the analysis of participant adequacy (KMO values) is deemed satisfactory for conducting data analysis in our study. Additionally, the study relied on self-reporting measures, and thus future research could employ a longitudinal design to explore the temporal dynamics of musical MM and SC. Future researchers can utilize a multi-method approach with more participants to capture a more comprehensive understanding of these constructs. Moreover, further investigations are needed to explore how musical MM works, what specific age characteristics can be identified, and what relationship can be established with SC and other related factors in different areas of music learning.

## 6. Conclusions

Overall, our study provides valuable insights into the dynamics between musical MM and musical SC within the context of music education. The results highlight the significant positive relationship between these constructs and shed light on the interplay among their subcomponents. Understanding these relationships can inform educational practices and interventions aimed at fostering students’ MM and SC in music education settings. In the comparison of students’ musical MM and musical SC by school level and gender, no significant differences were found between four different schools in the comparison of students’ musical SC, whereas higher significance levels were found in the comparison of their musical MM. Additionally, female students have higher levels than male students regarding both their musical MM and SC. Out of the six predicting variables such as gender, musical family background, usefulness of music lessons, music grade, elective instrumental training, and duration of instrumental training, only three variables (gender, musical family background, and usefulness of music lessons) were found to be predictive of students’ musical MM and musical SC. 

As for the practical implications, our findings contribute to the existing literature by emphasizing the positive relationship between musical MM and musical SC. The identification of significant factors with each construct provides insights into their underlying dimensions. These findings have implications for music educators, highlighting the importance of nurturing students’ MM and fostering a positive SC to promote their musical development. Regarding the comparison of students’ musical MM, it was found that students from School 4 have lower MM than other schools in our sample. This finding highlights a potential need for support or interventions to promote students’ MM. It is wise for educators and policy makers to note that factors such as gender, family musical background, music grades, resources, and the overall music culture may contribute to differences in students’ MM for music education. Our study also showed the findings of predicting the effects of background variables on students’ musical MM and SC. These results highlight the importance of creating inclusive and supportive environments in music education that consider gender differences, musical family background, and the usefulness of musical lessons and promote an understanding of the benefits of music lessons. Educators and policy makers should take into account these background variables when designing effective music education programs and interventions.

## Figures and Tables

**Table 1:** Factor loadings for EFA and convergent validity of the MM.

Items	Mean (SD)	Factor Loadings					Cronbach’s Alpha (>0.60) *	CR (>0.70) *	AVE (>0.50) *
		F1	F2	F3	F4	F5			
Rhy1	2.55 (1.37)	0.782					0.89	0.81	0.50
Rhy2	2.41 (1.38)	0.698							
Rhy3	2.34 (1.22)	0.692							
Rhy4	1.89 (1.07)	0.691							
Rhy5	2.38 (1.34)	0.623							
Rhy6	2.07 (1.99)	0.451							
Sing1	2.47 (1.37)		0.836				0.89	0.83	0.51
Sing2	2.32 (1.32)		0.792						
Sing3	2.41 (1.30)		0.661						
Sing4	2.38 (1.39)		0.656						
Sing5	2.96 (1.40)		0.553						
Sing6	2.41 (1.40)		0.513						
Read1	2.64 (1.34)			0.835			0.91	0.87	0.54
Read2	2.28 (1.26)			0.817					
Read3	2.20 (1.14)			0.786					
Read4	2.02 (1.16)			0.688					
Read5	2.32 (1.18)			0.665					
Read6	2.12 (1.11)			0.586					
Pleas1	3.50 (1.43)				0.879		0.91	0.90	0.75
Pleas2	3.18 (1.45)				0.838				
Pleas3	3.28 (1.38)				0.881				
Neg1	3.65 (1.43)					0.895	0.92	0.92	0.79
Neg2	3.72 (1.35)					0.893			
Neg3	3.67 (1.41)					0.890			
Total (24)	2.63 (0.91)						0.95	0.96	0.55

Note: * (recommended values).

**Table 2:** Factor loadings for EFA and convergent validity of the musical SC.

Items	Mean (SD)	Factor Loadings						Cronbach’s Alpha (>0.60) *	CR (>0.70) *	AVE (>0.50) *
		F1	F2	F3	F4	F5	F6			
Moo1	2.60 (1.12)	0.417						82	0.80	0.51
Moo2	3.31 (0.93)	0.556								
Moo3	3.02 (0.88)	0.689								
Moo4	2.63 (1.04)	0.697								
Moo5	3.19 (0.98)	0.781								
Moo6	2.67 (1.13)	0.497								
Com1	2.35 (0.97)		0.688					0.74	0.83	0.52
Com2	1.80 (0.83)		0.454							
Com3	1.72 (0.88)		0.649							
Com4	1.88 (0.95)		0.583							
Mus1	2.38 (1.39)			0.641				0.81	0.85	0.53
Mus2	1.73 (0.84)			0.703						
Mus3	1.74 (0.87)			0.818						
Mus4	2.68 (1.09)			0.782						
Mus5	2.14 (1.09)			0.695						
Dan1	2.23 (1.04)				0.518			0.73	0.80	0.51
Dan2	2.06 (1.17)				0.749					
Dan3	2.13 (1.19)				0.803					
Dan4	2.69 (1.16)				0.775					
Ide1	2.43 (1.07)					0.641		0.81	0.84	0.53
Ide2	2.26 (1.06)					0.703				
Ide3	2.15 (1.05)					0.812				
Ide4	1.99 (0.96)					0.782				
Ide5	2.05 (0.98)					0.695				
Ada1	2.06 (0.96)						0.792	0.79	0.80	0.50
Ada2	2.35 (1.09)						0.650			
Ada3	2.17 (1.07)						0.742			
Ada4	2.41 (1.08)						0.629			
Total (28)	2.63 (0.91)							0.91	0.96	0.51

Note: * (recommended values).

**Table 3:** Discriminant validity measures of both instruments.

Instruments	Component Correlation Matrix						
Musical MM	**Components**	**1**	**2**	**3**	**4**	**5**	
	1. Rhythm acquisition	0.707 *					
	2. Singing acquisition	0.648	0.714 *				
	3. Acquisition of Read/Knowledge	0.583	0.523	0.734 *			
	4. Mastery pleasures	0.430	0.347	0.394	0.866 *		
	5. Negative reaction to musical failure	0.532	0.433	0.465	0.368	0.888 *	
Musical SC	Components	1	2	3	4	5	6
	1. Mood management	0.714 *					
	2. Community	0.571	0.721 *				
	3. Musical ability	0.435	0.312	0.728 *			
	4. Movement and dance	0.453	0.518	0.214	0.714 *		
	5. Ideal musical self	0.360	0.315	0.273	0.277	0.728 *	
	6. Adaptive musical self	0.072	0.030	0.013	0.028	−0.096	0.707 *

Note: * Square root of AVE values.

**Table 4:** Correlation between musical MM and SC.

Instruments	Musical SC						
	MM		COM	MA	MD	IMS	AMS
Musical MM	RP	0.37	0.53	0.42	0.37	0.65	0.52
	SP	0.46	0.50	0.46	0.40	0.66	0.58
	PRK	0.35	0.52	0.39	0.29	0.64	0.50
	MP	0.29	0.38	0.28	0.39	0.55	0.46
	NRF	0.06	0.12	0.30	0.12	0.32	0.23

Note: MM (mood management), COM (community), MA (musical ability), MD (movement and dance), IMS (ideal musical self), AMS (adaptive musical self) for the musical SC inquiry; RP (rhythmic persistence), SP (singing persistence), PRK (musical reading and knowledge acquisition), MP (musical pleasure), NRF (negative reaction to failure) for the musical MM inquiry.

**Table 5:** Correlation of background variables with musical MM and SC.

Variables	RP	SP	RNP	MP	NRF	Gen	MG	EIT	DIT	UML	MFB
MM	0.374 **	0.457 **	0.357 **	0.287 **	0.062	0.308 **	0.139	0.175 *	0.136	0.326 **	0.346 **
Com	0.530 **	0.504 **	0.518 **	0.381 **	0.115	0.428 **	0.182 *	0.284 **	0.305 **	0.427 **	0.263 **
MA	0.418 **	0.463 **	0.391 **	0.281 **	0.297 **	0.190 *	0.284 **	0.339 **	0.409 **	0.307 **	0.290 **
MD	0.372 **	0.404 **	0.286 **	0.393 **	0.122	0.490 **	0.154	0.077	0.049	0.416 **	0.132
IMS	0.647 **	0.663 **	0.640 **	0.551 **	0.321 **	0.401 **	0.016	0.216 *	0.172 *	0.622 **	0.229 **
AMS	0.522 **	0.584 **	0.496**	0.455 **	0.232 **	0.442 **	0.117	0.155	0.131	0.362 **	0.276 **
Gen	0.384 **	0.488 **	0.329 **	0.337 **	0.175 *	1	0.271 **	0.204 *	0.140	0.372 **	0.202*
MG	0.175 **	0.154	0.243 **	0.172 *	0.138		1	0.176	0.262 *	0.175	0.169
EIT	0.272 **	0.265 **	0.340 **	0.142	0.121			1	0.828 **	0.218 **	0.218 **
DIT	0.221 **	0.191 *	0.305 **	0.132	0.112				1	0.148 *	0.279 **
UML	0.563 **	0.609 **	0.579 **	0.463 **	0.277 **					1	0.164
MFB	0.283 **	0.390 **	0.267 **	0.160	0.146						1

Note: ** *p* < 0.01, * *p* < 0.05 RP (rhythmic persistence), SP (singing persistence), PRK (musical reading and knowledge acquisition), MP (musical pleasure), NRF (negative reaction to failure) for the musical MM inquiry; MM (mood management), Com (community), MA (musical ability), MD (movement and dance), IMS (ideal musical self), AMS (adaptive musical self) for the musical SC inquiry; background variables—Gen (gender), MG (music grade), EIT (elective instrumental training), DIT (duration of instrumental training, UML (usefulness of music lessons), MFB (music family background).

## Data Availability

Data are unavailable due to privacy or ethical restrictions.

## References

[1] Mark M.L. (2005). Why does our profession need advocacy?. Int. J. Music Educ..

[2] Fiedler D., Hasselhorn J. (2020). The relationship between musical self-concept and motivation in music education. Rel. Bull. Empir. Music Educ. Res..

[3] Józsa K., Kis N., Barrett K.C. (2019). Mastery motivation, parenting and school achievement among Hungarian adolescents. Eur. J. Psychol. Educ..

[4] Morgan G.A., MacTurk R.H., Hrncir E.J., MacTurk R.H., Morgan G.A. (1995). Mastery motivation: Overview, definitions, and conceptual issues. Mastery Motivation: Origins, Conceptualizations, and Applications.

[5] Calchei M., Oo T.Z., Józsa K. (2023). Subject specific mastery motivation in Moldovan middle school students. Behav. Sci..

[6] Barrett K.C., Morgan G.A., Elliot A. (2018). Mastery Motivation: Retrospect, present, and future directions. Advances in Motivation Science.

[7] Józsa K., Kis N., Huang S.-Y. (2017). Mastery motivation in school subjects in Hungary and Taiwan. Hung. Educ. Res. J..

[8] Jόzsa K., Molnár É.D., Barrett K.C., Fox N.A., Morgan G.A., Fidler D.J., Daunhauer L.A. (2013). The relationship between mastery motivation, self-regulated learning and school success: A Hungarian and European perspective. Handbook on Self-Regulatory Processes in Development: New Directions and International Perspectives.

[9] Borbélyová D. (2017). Az iskolai motiváció formálódása az első évfolyamba való beilleszkedés kontextusában. Zborník Medzinárodnej Vedeckej Konferencie Univerzity J. Selyeho-2017:”Hodnota, Kvalita A Konkurencieschopnosť-Výzvy 21. Storočia”-Sekcie Pedagogických Vied.

[10] Comeau G., Huta V., Lu Y., Swirp M. (2019). The Motivation for Learning Music (MLM) questionnaire: Assessing children’s and adolescents’ autonomous motivation for learning a musical instrument. Motiv. Emot..

[11] Miksza P., Evans P., McPherson G.E. (2021). Motivation to pursue a career in music: The role of social constraints in university music programs. Psychol. Music.

[12] Mulder M., Hitters E. (2021). Visiting pop concerts and festivals: Measuring the value of an integrated Live Music Motivation Scale. Cult. Trends.

[13] Wieser M., Müller F.H. (2022). Motivation in instrumental music instruction before and during the remote learning phase due to COVID-19 crisis. Music Sci..

[14] Morin A.J.S., Scalas L.F., Vispoel W., Marsh H.V., Wen Z. (2016). The Music Self-Perception Inventory: Development of short form. Psychol. Music.

[15] Szenczi B., Kis N., Józsa K. (2018). Academic self-concept and mastery motivation in students with learning disabilities. J. Psychol. Educ. Res..

[16] Mawang L.L., Kigen E.M., Mutweleli S.M. (2019). The relationship between musical self-concept and musical creativity among secondary school music students. Int. J. Music Educ..

[17] Mazur I., Hrinchenko T., Teplova O., Onofrichuk L., Priadko O. (2022). Cognitive determination of musical thinking and musical self-concept of students and musicians: Comparative diagnostics, aspects of modeling and forecasting. Harmon. J. Arts Res. Educ..

[18] Demorest M., Kelley J., Pfordresher P.Q. (2017). Singing ability, musical self-concept and future music participation. J. Res. Music Educ..

[19] Hallam S. (2002). Musical Motivation: Towards a Model Synthesising the Research.

[20] Schmidt C.P. (2005). Relations among motivation, achievement, and music experience variables in secondary instrumental music students. J. Res. Music. Educ..

[21] Spychiger M., Gruber L., Olbertz F., Louhivuori J., Eerola T., Saarikallio S., Himberg T., Eerola P.-S. (2009). Musical self-concept-presentation of a multidimensional model and its empirical analyses. Proceedings of the 7th Triennial Conference of European Society for the Cognitive Sciences of Music (ESCOM 2009).

[22] Arens A.K., Fiedler D., Hasselhorn J. (2022). Embedding the self-concept in music as a school subject into academic self-concept research. Z. Fur Erzieh..

[23] Ryan R.M., Deci E.L. (2000). Self-determination theory and the facilitation of intrinsic motivation, social development, and well-being. Am. Psychol..

[24] Legault L. (2020). Self-Determination Theory. Encyclopedia of Personality and Individual Differences.

[25] Tang W.G., Vandenberghe C. (2020). The reciprocal relationship between affective organizational commitment and role overload: When autonomy need satisfaction meets the individual self-concept. J. Occup. Organ. Psychol..

[26] Nazario L. (2022). da C. Freedom as a trigger for musical creativity. Res. Stud. Music Educ..

[27] Evans P., Liu M.Y. (2019). Psychological needs and motivational outcomes in a high school orchestra program. J. Res. Music Educ..

[28] White R. (1959). Motivation reconsidered: The concept of competence. Psychol. Rev..

[29] Yarrow L.J., Morgan G.A., Jennings K.D., Harmon R.J., Gaiter J.L. (1982). Infants’ persistence at tasks: Relationships to cognitive functioning and early experience. Infant Behav. Dev..

[30] Yarrow L.J., Rubenstein J.L., Pedersen F.A. (1975). Infant and Environment: Early Cognitive and Motivational Development.

[31] Barrett K.C., Morgan G.A., MacTurk R.H., Morgan G.A. (1995). Continuities and discontinuities in mastery motivation in infancy and toddlerhood: A conceptualization and review. Mastery Motivation: Origins, Conceptualizations and, Applications.

[32] Elliot A.J., Elliot A., Dweck C. (2005). A conceptual history of the achievement goal construct. Handbook of Competence and Motivation.

[33] Wang J., Barrett K.C., Barrett K.C., Fox N.A., Morgan G.A., Fidler D.J., Daunhauer L.A. (2013). Mastery motivation and self-regulation during early childhood. Handbook of Self-Regulatory Process in Development: New Directions and International Perspectives.

[34] Morgan G.A., Józsa K., Liao H.-F., Morgan G.A., Liao H.-F., Józsa K. (2020). Overview of mastery motivation, assessment, and this book. Assessing Mastery Motivation in Children Using the Dimensions of Mastery Questionnaire (DMQ).

[35] Janurik M., Kis N., Szabó N., Józsa K. (2021). Az ének-zene tantárgy iránti attitűd összefüggése az iskolai zenetanulás iránti motivációval hetedik osztályos tanulók körében. [The relationship of the music attitude of grade 7 students with musical MM and some background variables]. Neveléstudomány.

[36] Harter S., Leary M.R., Tangney J.P. (2003). The development of self-representations during childhood and adolescence. Handbook of Self and Identity.

[37] Shavelson R.J., Hubner J.J., Stanton G.C. (1976). Self-concept: Validation of construct interpretations. Rev. Educ. Res..

[38] Hattie J. (1992). Self-Concept.

[39] Marsh H.W. (1984). Relationships among dimensions of self-attribution. dimensions of self-concept and academic achievements. J. Educ. Psychol..

[40] Marsh H.W. (1990). The structure of academic self-concept: The Marsh/Shavelson Model. J. Educ. Psychol..

[41] Brunner M., Keller U., Dierendonck C., Reichert M., Ugen S., Fischbach A., Martin R. (2010). The structure of academic self-concepts revisited: The nested Marsh/Shavelson model. J. Educ. Psychol..

[42] Gogol K., Brunner M., Preckel F., Goetz T., Martin R. (2016). Developmental dynamics of general and school-subject-specific components of academic self-concept, academic interest and academic anxiety. Front. Psychol..

[43] Green J., Martin A.J., Marsh H.W. (2007). Motivation and engagement in English, Mathematics and Science high school subjects: Towards an understanding of multidimensional domain specificity. Learn. Individ. Differ..

[44] Spychiger M., Macdonald R., Hargreaves D.J., Miell D. (2017). From musical experience to musical identity. Handbook of Musical Identities.

[45] Vispoel W.P. (1995). Self-concept in artistic domains: An extension of the Shavelson model. J. Educ. Psychol..

[46] Austin J.R., Vispoel W.P. (1998). How American adolescents interpret success and failure in classroom music: Relationships among attributional beliefs, self-concept, and achievement. Psychol. Music.

[47] Vispoel W.P., Marsh H.W., Craven R.G., McInerney D.M. (2003). Measuring and understanding self-perceptions of musical ability. International Advances in Self Research.

[48] Vispoel W.P., Craven R., Marsh W.H. (2000). Music self-concept: Instrumentation, structure, and theoretical linkages. Self-Concept Theory. Research and Practice: Advances for the Millennium.

[49] Marsh H.W. (1993). Relations between global and specific domains of self: The importance of individual importance, certainty, and ideals. J. Personal. Soc. Psychol..

[50] Sandene B.A. (1997). An investigation of variables related to student motivation in instrumental music. Dissertation Abstracts International.

[51] Austin J.R. (1988). The Effect of music contest format on self-concept, motivation, achievement and attitude of elementary band students. J. Res. Music Educ..

[52] Marsh H.W., Craven R.G., Hinkley J.W., Debus R.L. (2003). Evaluation of the big-two factor theory of academic motivation orientations: An evaluation of jingle-jangle fallacies. Multivar. Behav. Res..

[53] Fiedler D., Spychiger M. (2017). Measuring “Musical Self-Concept” throughout the years of adolescence with MUSCI-youth: Validation and adjustment of the Musical Self-Concept Inquiry (MUSCI) by investigating samples of students at secondary education schools. Psychomusicology Music Mind Brain.

[54] (2020). Kerettanterv az Általános Iskola 5–8. Évfolyama Számára. Ének-Zene. [Framework Curriculum for Grade 5–8 Students. Music.]. https://www.oktatas.hu/kozneveles/kerettantervek/2020_nat/kerettanterv_alt_isk_5_8.

[55] Göktürk Cary D. (2012). Kodály and Orff: A Comparison of Two Approaches in Early Music Education. Uluslar. Yönetim Iktisat Ve Islet. Derg..

[56] Choksy L. (1999). The Kodály Method I: Comprehensive Music Education.

[57] Dobszay L. (2009). After Kodály: Reflections on Music Education.

[58] Borbélyová D. (2021). A Pedagógiai Diagnosztika Új Útjai És Kihívásai. [New Ways and Challenges of Pedagogical Diagnostics].

[59] Kline R.B. (2016). Principles and Practices of Structural Equation Modeling.

[60] Boone W.J. (2016). Rasch-analysis for instrument development: Why, when, and how?. CBE Life Sci. Educ..

[61] Gliner J.A., Morgan G.A., Leech L.N. (2017). Research Methods in Applied Settings: An Integrated Approach to Design and Analysis.

[62] Davies T.M. (2016). Theology. The Book of Ruth.

[63] Chua Y.P. (2023). A Step-By-Step Guide Pls-Sem Data Analysis Using SmartPLS4.

[64] Tsaousis I., Alghamdi M.H. (2022). Examining academic performance across gender differently: Measurement invariance and latent mean differences using bias-corrected bootstrap confidence intervals. Front. Psychol..

[65] Marsh H.W. (1986). Global self-esteem: Its relation to specific facets of self-concept and their importance. J. Personal. Soc. Psychol..

[66] Józsa K., Barrett K.C. (2018). Affective and social mastery motivation in preschool as predictors of early school success: A longitudinal study. Early Child. Res. Q..

[67] Somers C.L., Gill-Scalcucci S., Flett G.L., Nepon T. (2022). The utility of brief mattering subscales for adolescents: Associations with learning motivations, achievement, executive function, hope, loneliness, and risk behavior. J. Psychoeduc. Assess..

[68] Wang P.J., Hwang A.W., Liao H.F., Chen P.C., Hsieh W.S. (2011). The stability of mastery motivation and its relationship with home environment in infants and toddlers. Infant Behav. Dev..

[69] Sanders P.D., Browne L.A. (1998). Music Self-Concept of Non-Music Majors. Contrib. Music Educ..

[70] Clements A.C. (2002). The Importance of Selected Variables in Predicting Student Participation in Junior High Choir. Ph.D. Thesis.

[71] Shonkoff J.P., Phillips D.A. (2000). From Neurons to Neighborhoods: The Science of Early Childhood Development.

[72] Siebenaler D.J. (2006). Factors that predict participation in choral music for high-school students. Res. Issues Music Educ..

